# Errors in Figure, Text, and Table

**DOI:** 10.1001/jamanetworkopen.2024.34909

**Published:** 2024-08-23

The Original Investigation “P2Y12 Inhibitor Monotherapy vs Dual Antiplatelet Therapy After Deployment of a Drug-Eluting Stent: The SHARE Randomized Clinical Trial,”^1^ published March 7, 2024, has been corrected to fix an error in Figure 3, in which the row labels “men” and “women” were transposed. The same error appeared in the main text; the corrected sentence now reads, “P2Y12 inhibitor monotherapy was more favored over DAPT in men than in women (*P* = .04 for interaction).” In Table 2, only clinical outcomes between 3 and 12 months after the index percutaneous coronary intervention were studied, so the number of patients in the P2Y12 inhibitor monotherapy group has been changed from 694 to 674 and the number of patients in the dual antiplatelet therapy group has been changed from 693 to 673. This article was previously corrected on April 24, 2024, to fix an error in Figure 1.^2^
